# Keratin 5 expression defines a niche-supported hierarchy in tubo-ovarian cancer

**DOI:** 10.47248/chp2603020006

**Published:** 2026-05-12

**Authors:** Zhiyu Tian, Karen McLean

**Affiliations:** 1 Department of Pharmacology & Therapeutics, Roswell Park Comprehensive Cancer Center, Buffalo, NY 14203, USA; 2 Department of Gynecologic Oncology, Roswell Park Comprehensive Cancer Center, Buffalo, NY 14203, USA

## Abstract

Tubo-ovarian high-grade serous carcinoma (HGSC) is characterized by extreme cellular heterogeneity. A tumorigenic subset of cancer-propagating cells (CPC) with stem-like properties coexists with a large population of non-cancer-propagating cells (non-CPC), but the functional interplay between these two cell types remains poorly understood. In this issue, Bidarimath et al. identify keratin 5 (KRT5) as a marker of CPC in HGSC and propose a niche model in which osteopontin (OPN) sustains KRT5-positive CPC through interactions with KRT5-negative, non-tumorigenic cells. By combining in vitro, in vivo, and patient-derived data, the study links a supportive tumor microenvironment to the maintenance of a phenotypically defined CPC population.

Cellular heterogeneity is a hallmark of tubo-ovarian high-grade serous carcinoma (HGSC), with only a subset of tumor cells possessing stem-like long-term self-renewal capacity that drives tumor growth, while the bulk population lacks sustained tumorigenic potential [1,2]. Multiple CPC markers have been reported for HGSC, including CD44, CD133, and ALDH1 [1]. In tubo-ovarian cancer, such CPC populations have been implicated in recurrence and chemoresistance, with their maintenance closely linked to signals from the tumor microenvironment [1]. However, the functional relationships among tumor cell subpopulations remain incompletely defined.

In this issue, Bidarimath and colleagues identified KRT5 as a marker of CPC in HGSC [3]. While previous studies have associated KRT5 expression with chemoresistance [4,5], the present study moves beyond correlation and establishes the role of KRT5 as a CPC marker through a series of functional assays. KRT5-positive cells display sustained organoid forming capacity in vitro, tumor initiating potential *in vivo*, and marked resistance to standard chemotherapeutics. The study also advances the role of the non-CPC compartment. Rather than being passive byproducts of differentiation, KRT5-negative cells act as active participants in tumor maintenance. Transcriptomic profiling reveals that KRT5-negative cells are enriched for secretory programs, most notably the expression of the *SPP1* gene, encoding OPN (Figure 1), a secreted glycoprotein known to regulate stem cell function and tumor progression through receptors such as CD44 and integrins [6,7]. Functional assays convincingly demonstrate that OPN increases KRT5-positive CPC, enhances organoid growth, and confers chemoresistance, while its depletion sensitizes tumors to cisplatin. The novelty of this study is that this pro-CPC niche is not stromal or immune-derived but instead generated by differentiated epithelial tumor cells. This hierarchy simultaneously produces both propagating cells and the signals required to sustain them (Figure 1).

Such a model carries important therapeutic implications. Efforts to eradicate cancer stem cells have historically been limited by incomplete responses and rapid relapse, often attributed to plasticity or incomplete targeting [8]. Importantly, the clinical relevance of KRT5 as a CPC marker is demonstrated in this study, with the finding that a higher frequency of KRT5-positive cells was associated with a shorter overall survival in a cohort of 470 patients with tubo-ovarian HGSC. The present study suggests that even if the bulk of CPC are effectively targeted, the persistence of a supportive niche may enable residual cells to survive or re-emerge. Conversely, disrupting niche signals such as OPN may sensitize CPC to conventional therapies, offering a combinatorial strategy that targets both cell-intrinsic and microenvironmental dependencies.

At the same time, several questions remain unsolved. The role of CD44 as a potential CPC marker in the system studied and its potential overlapping expression with KRT5 is underexplored. Additionally, confirmatory studies in additional model systems beyond the SKOV3 cell line and the included patient derived organoids will be important to further strengthen the reported findings in high-grade serous carcinoma [9].

Overall, this manuscript by Bidarimath and colleagues adds to and advances our understanding of CPC maintenance in tubo-ovarian HGSC by defining a novel role for OPN secreted by KRT5-negative cells in supporting KRT5-positive CPC. Thus, therapeutic approaches to successfully eradicate these cancers may require agents that target not only the CPC population of tumor cells but also the microenvironment including differentiated epithelial tumor cells that supports CPC self-renewal.

## Figures and Tables

**Figure 1.**
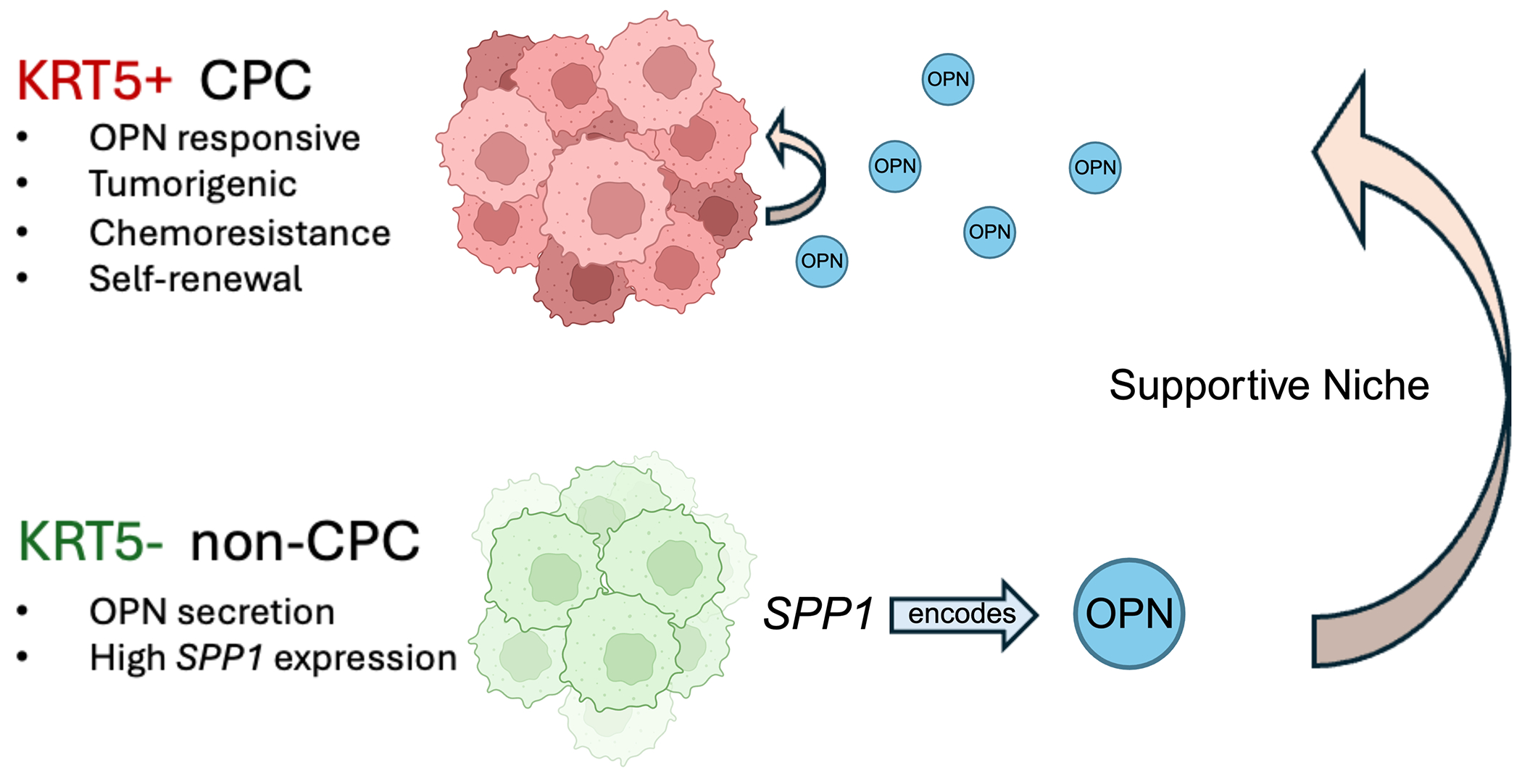
KRT5-positive CPC population gives rise to KRT5-negative, non-CPC progeny. KRT5-negative, non-CPC cells exhibit high expression of *SPP1*, which encodes OPN, thereby contributing to a supportive niche that sustains the self-renewal and tumorigenic capacity of KRT5-positive CPC. Created with BioRender.com.

## References

[1] Alizadeh H, Akbarabadi P, Dadfar A, Tareh MR, Soltani B. A comprehensive overview of ovarian cancer stem cells: correlation with high recurrence rate, underlying mechanisms, and therapeutic opportunities. Mol Cancer. 2025;24:135. 10.1186/s12943-025-02345-3 40329326 PMC12057202

[2] Clevers H. The cancer stem cell: premises, promises and challenges. Nat Med. 2011;17(3):313–319. 10.1038/nm.2304 21386835

[3] Bidarimath M, Ralston CQ, Bidarimath N, Rose IM, Colina D, Schmoeckel E, Keratin 5 marks cancer propagating cells sustained by an osteopontin-producing niche in high-grade serous ovarian carcinoma. Cancer Heterog Plast. 2026;3(2):0005. 10.47248/chp2603020005 42111394 PMC13155427

[4] Corr BR, Finlay-Schultz J, Rosen RB, Qamar L, Post MD, Behbakht K, Cytokeratin 5 positive cells represent a therapy resistant subpopulation in epithelial ovarian cancer. Int J Gynecol Cancer. 2015;25(9):1565–1573. 10.1097/IGC.0000000000000553 26495758 PMC4635519

[5] Ricciardelli C, Lokman NA, Pyragius CE, Ween MP, Macpherson AM, Ruszkiewicz A, Keratin 5 overexpression is associated with serous ovarian cancer recurrence and chemotherapy resistance. Oncotarget. 2017;8(11):17819–17832. 10.18632/oncotarget.14867 28147318 PMC5392289

[6] Zhao H, Chen Q, Alam A, Cui J, Suen KC, Soo AP, The role of osteopontin in the progression of solid organ tumour. Cell Death Dis. 2018;9(3):356. 10.1038/s41419-018-0391-6 29500465 PMC5834520

[7] Pietras A, Katz A, Ekström E, Wee B, Halliday J, Pitter K, Osteopontin-CD44 Signaling in the Glioma Perivascular Niche Enhances Cancer Stem Cell Phenotypes and Promotes Aggressive Tumor Growth. Cell Stem Cell. 2014;14(3):357–369. 10.1016/j.stem.2014.01.005 24607407 PMC3999042

[8] Mai Y, Su J, Yang C, Xia C, Fu L. The strategies to cure cancer patients by eradicating cancer stem-like cells. Mol Cancer. 2023;22:171. 10.1186/s12943-023-01867-y 37853413 PMC10583358

[9] Adams KM, Wendt J-R, Wood J, Olson S, Moreno R, Jin Z, Cell-intrinsic platinum response and associated genetic and gene expression signatures in ovarian cancer. Cancer Gene Ther. 2025;32:985–996. 10.1038/s41417-025-00941-5 40683954 PMC12396967

